# UAV Positioning for Throughput Maximization Using Deep Learning Approaches

**DOI:** 10.3390/s19122775

**Published:** 2019-06-20

**Authors:** Yirga Yayeh Munaye, Hsin-Piao Lin, Abebe Belay Adege, Getaneh Berie Tarekegn

**Affiliations:** 1 Department of Electrical Engineering and Computer Science, National Taipei University of Technology, Taipei 10608, Taiwan; abbblybelay@gmail.com (A.B.A.); gechb21@gmail.com (G.B.T.); 2 Department of Electronic Engineering, National Taipei University of Technology, Taipei 10608, Taiwan; hplin@ntut.edu.tw

**Keywords:** user throughput, maximization, UAV, positioning, deep learning (DL)

## Abstract

The use of unmanned aerial vehicles (UAVs) as a communication platform has great practical importance for future wireless networks, especially for on-demand deployment for temporary and emergency conditions. The user throughput estimation in a wireless system depends on the data traffic load and the available capacity to support that load. In UAV-assisted communication, the position of the UAV is one major factor that affects the capacity available to the data flows being served. This study applies multi-layer perceptron (MLP) and long short term memory (LSTM) approaches to determine the position of a UAV that maximizes the overall system performance and user throughput. To analyze and evaluate the system performance, we apply the hybrid of MLP-LSTM for classification regression tasks and K-means algorithms for automatic clustering of classes. The implementation of our work is done through TensorFlow packages. The performance of our proposed system is compared with other approaches to give accurate and novel results for both classification and regression tasks of the user throughput maximization and UAV positioning. According to the results, 98% of the user throughput maximization accuracy is correctly classified. Moreover, the UAV positioning provides accuracy levels of 94.73%, 98.33%, and 99.53% for original datasets (scenario 1), reduced features on the estimated values of user throughput at each grid point (scenario 2), and reduced feature datasets collected on different days and grid points achieved maximum throughput (scenario 3), respectively.

## 1. Introduction

Recently, UAVs, which are also known as drones, have received interest from different scholars. UAVs may be used in different situations, such as for security issues, disasters, and remote areas as well as on sport fields [1,2]. Nowadays, the growth and advancement of wireless mobile communication technology has a great influence on citizens’ day-to-day activities. This leads mobile users to demand more bandwidth and have greater interest in wireless communication at any time and place. Of course, to satisfy the interest of mobile users, concerned organizations have attempted to expand the coverage of long term evolution (LTE) and access points (APs) based on wireless fidelity (Wi-Fi) connections. However, their large consumption and the fixed location do not solve the problem well for remote areas [3,4,5]. Thus, the use of UAVs as aerial base stations (BSs) as a communication platform is important for the assistance of territorial networks. This is a vital area of enhancement for contemporary technology as a key stage for fifth-generation (5G) networks and has been a main focus of researchers and service providers [6,7]. For instance, UAVs are deployed for basic wireless data collection issues for remote areas and emergency situations as well as to enhance the capacity of the wireless communication network in terrestrial hotspots [8,9,10,11,12]. In addition, UAVs can be utilized as aerial communication BSs for ground users to deliver air-to-ground (A2G) access link services. APs in the sky are also used for information dissemination and data collection from the ground (e.g., sensors and actuators in Internet-of-Things (IoT) networks) [12,13]. Indeed, the use of UAVs as aerial BSs provides several advantages compared with terrestrial BSs. Due to the higher altitude and distance coverage capability, aerial BSs have a higher chance of having line-of-sight (LoS) to ground users. In addition, UAVs can have easy movement and flexible deployment, and they use antennas for further capacity enhancement. Hence, they can provide a rapid on-demand service [5]. 

The main modifications of conventional terrestrial wireless communications and UAV-assisted network services can be compared. First, UAV-assisted aerial communication platforms can be quickly deployed based on user data access request/demand, and they have a cost minimization benefit as well as being preferable for emergency scenarios. However, terrestrial wireless infrastructures are highly vulnerable to natural disasters, because their deployment is from a fixed location. Second, the A2G access link between UAVs with ground users has much stronger LoS links than conventional ground-to-ground (G2G) links [12]. As a result, the UAV-assisted aerial BSs are expected to provide better wireless coverage and higher user throughput capacity than ground BSs. Additionally, due to the fully controllable mobility of three-dimensional (3D) space, UAVs can adaptively change their locations over time to reduce the height and distance relative to the ground users; this leads to further improvement of other wireless network performances [9]. Third, the signal distribution and delivery based on UAVs cannot be largely affected by channel impairments, such as shadowing and fading [10]. Fourth, UAVs can be deployed more flexibly and moved more freely in the 3D space. Therefore, these identical characteristics yield both opportunities and challenges in designing UAV-enabled wireless communications, which cannot be reachable with conventional terrestrial systems or immovable BSs [14]. Nevertheless, there is also an open challenge that needs to be solved for UAV-enabled communications, such as [15] UAV positioning of communications for resource saving and user throughput maximization. 

The study in [16] was motivated by the issue of A2G access links between UAVs and ground user connections. The UAV-assisted aerial communication stages would be performed through random distribution and fully mobile communication. In the case of random distribution of UAV communication, previous works [16,17,18,19,20,21,22] considered the optimization of deployment locations to enhance network performance. For instance, considered the two-dimensional (2D)/3D placement of UAVs to increase the network coverage (i.e., to serve users by UAVs based on the quality-of-service (QoS) requirement) and minimize the number of UAVs required to maintain wireless coverage [23,24]. The author in [19] optimized the flying altitude of UAVs to maximize user throughput for multiuser communications with the consideration of adjustable directional antennas with UAVs. Furthermore, analyzed the average performance of random communication of UAV-enabled networks through the implementation of stochastic geometry theory [16,17]. For the fully mobile communication of UAVs, existing works, such as [25,26,27], proposed the control of a dynamic location within a specified period of time for the enhancement of user communication rates. These studies assumed that the UAV-enabled mobile communication could adapt to and control the tasks of trajectory systems through wireless resource allocation for the purpose of maximizing the end-to-end communication between the transmitter (Tx) and receiver (Rx). In [28,29], the movability of UAVs was used to enhance the network connectivity and a field experiment was conducted with UAV-assisted communication in which the user throughput maximization problem was studied by optimizing the source of power allocations with consideration of time [30,31]. 

To the best of our knowledge, these issues are still open problems that have not been deeply investigated yet. The MLP-LSTM based approach is the first system that jointly structures or models user data and UAV-based OFDM signals in the real environment. Note that related work was conducted in [21], where the multi-hop wireless backhauls were used to communicate between ground BSs and the main network, and the group of UAVs was designed to maximize the usefulness of the backhaul network being optimized. To fill such research gaps, we consider three UAVs connected through wireless multi-hop backhauls with the core network. Under this layout, we set an objective to obtain the positioning of UAVs to achieve user throughput performance maximization. Different from previous works, our study focuses on both A2G and air-to-air (A2A) access links as well as applying deep learning (DL) approaches for system design and prediction. In addition, we use the real environment for data collection and reduce the dataset dimensionality based on linear discriminant analysis (LDA) approaches. The integration of LDA and long short term memory (LSTM)-multi-layer perceptron (MLP) reduces the size of large datasets and generates a more accurate positioning system. It also improves computational times by reducing space usage from the complex into simpler features [32]. This motivated us to explore the problem based on DL approaches, because it is an efficient approach with the smallest number of training and testing errors. Thus, it is a better way to assess a large number of datasets as well as measure the accuracy of larger data collection environments than traditional machine learning (ML) approaches. 

As compared to traditional ML approaches, DL approaches improve the accuracy of regression and classification tasks in different fields, such as image recognition, signal processing, and speech recognition [33]. In addition, they allow features to be extracted automatically from a given dataset and outperform ML techniques, especially if the dataset size is large and applied for compound issues [32]. Therefore, the results show that the proposed algorithms for UAV positioning and user throughput maximization can easily enhance the network performance for each location of users. Regarding the reliability of the distribution of orthogonal frequency-division multiplexing (OFDM) signal for a large number of users, it reduces the time complexity and improves the signal transmission to ground users. We propose an iterative weighted interpolation positioning (IWIP) method for measuring the distance between ground users and the UAV location scheme based on OFDM signal transmission for the A2G access link. This method can be applied for the purpose of multi-user localization. It performs effectively, generates high positioning accuracy [32], and it contains both the merits and demerits of centralized and decentralized positioning systems.

### LSTM Learning Architecture for Our System

**Figure 1:**
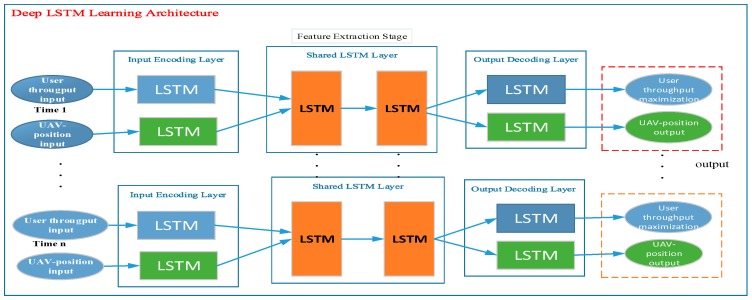
Illustration of long short term memory (LSTM) architecture for our system.

According to [33], the LSTM, or memory network, is composed of memory blocks. The memory cells contain self-connections for storing and remembering. The temporal state of the network and gates control the flow of information, and an input gate controls the flow of input activity into the memory cell. An output gate controls the output flow of cell activity into the rest of the network and a forget gate to be forgotten from the previous cell state. Based on these special structures, the LSTM approach has the capacity to learn and forecast dynamic systems [34,35], and to learn long-term dependencies, which cannot be done using other ML or DL approaches. Based on these advantages, we are interested in applying LSTM as the main algorithm for training, model construction, and predictions of our proposed methods to the analysis and maximization of throughput of UAV-based aerial networks. Different scholars have proposed ML approaches, such as the artificial neural network (ANN) and K-nearest neural network (KNN), but they have shown poor learning capacity [32], compared with the LSTM algorithm. This poor learning capacity does not enable a system, which can adopt a real and dynamic environment, to be created. Therefore, to improve the learning performance, we propose the use of the MLP-LSTM hybrid, and we compare it to other algorithms. As the advanced version of the recurrent neural network (RNN) [33], LSTM has been demonstrated to be an efficient approach for modeling sequential data, like text [34]. In order to improve the performance of user throughput based on UAV positioning, we collected a large number of real datasets. As shown in Figure 1, we structured the general layout of the LSTM algorithm for applying prediction tasks to our system design. The deep LSTM learning architecture contains time variance (the user throughput value and the location of UAV) as the input layer, a shared LSTM layer (feature extraction stage), and user throughput maximization, which is delivered through the output layer. 

In this study, we built a DL-based system for prediction and regression tasks of the UAV-based communication network to maximize user throughput (as shown in Figure 1). The learning components of our system were designed through the LSTM learning architecture that contains the following components: (1) A time series (which contains the user throughput and UAV positioning dataset values), (2) an encoding layer which is applied for the input of encoding layer sequences, (3) the hidden layer or shared layer part (mostly performs feature extraction tasks), and (4) a decoding layer that allows the output sequences to generate the final results for user throughput maximization and UAV positioning. The proposed learning architecture is able to jointly learn the user data from the grid points and UAV altitudes as well as determine the distance from the data source and permits to learn the input sequences with different time scales. Regardless of the locations, the number of users, time periods, distance of users and UAVs, and the impact of LoS/non-line-of-sight (NLoS) characteristics, the proposed LSTM learning architecture can automatically learn and predict a large number of user locations and their A2G access links with the UAV location. 

The main contribution of our work is the estimation of the UAV position in order to maximize the A2G link access coverage performance with the use of a minimum transmitting power and the maximization of user throughput. This led us to apply the method to different environment types, such as sub-urban, urban, and rural areas, because the future 5G network will contain a heterogeneous wireless network and highly loaded user interest to get a highly accessible bandwidth in wireless networks. With this consideration, we collected our datasets from the real environment, which has urban and sub-urban environment characteristics. We selected a real data collection area with ground mobile users and UAVs that use non-directional antennas. To perform and design the proposed system, we proposed DL approaches (i.e., MLP and LSTM) to train and predict the improvement of user throughput based on the efficient estimation of UAV positioning. First, we derived the A2G link access coverage probability considering the UAV altitude, Los/NLoS, elevation angle, received signal strength (RSS), signal-noise-ratio(SNR), and user-to-user distances. Next, we applied a K-means algorithm to cluster the data collection environment into three classes based on their signal variation and LoS/NLoS (as classes 1, 2, and 3). Our results show that considering the size of the desired area, the number of available UAVs, the distance between the UAV to the users, and the altitude or location of the UAVs have great impacts on the maximization of user throughput, a benefit that is shared by all users. Generally, after analyzing the above facts and related works, our contributions can be summarized as follows:

(1) DL (i.e., MLP and LSTM) approaches are proposed to better predict the positions of UAVs and for user throughput maximization. The optimal positioning of UAVs was evaluated based on the A2G access link, the power usage, and the data flows.
(2) We proposed a K-means algorithm for the classification of our data collection environment into classes based on their signal strengths, and we used LDA for dataset dimensionality reduction and iterative weighted interpolation positioning (IWIP) for computing each user’s distance. Furthermore, to maximize user throughput, we considered the positioning of a UAV based on the data rate request and positions of its connected users.
(3) Finally, our work introduces a simple and computationally efficient method for keeping several mobile users within the transmission range of the UAVs in the dynamic environment while determining a UAV position that maximizes throughput; previous researches did not pay much attention to these factors. The rest of the paper is structured as follows. Section 2, contains related works, Section 3, discusses data collection and the proposed system. Section 4, reports the experimental results and discussions. The last section summarizes the major conclusions and references.

## 2. Related Works

Currently, UAVs have been adopted in several sectors, such as weather monitoring and traffic control [35], and the application of UAVs in wireless communication systems has been given excessive consideration [2,3,4,5,6,7,8,9,10,11]. In contrast, conventional networks are deployed in a fixed manner, APs are immovable from their given locations, but deployed UAV-assisted wireless networks are flexible and advantageous in terms of cost. In [35], the concept of user throughput maximization issues was introduced for the purpose of optimizing time resource allocation through wireless-powered communication networks (WPCNs). The authors of [36] used an algorithm to improve the minimum user throughput performance based on several antennas and time allocation algorithms, and an analysis of user throughput maximization with a full-duplex hybrid access point (HAP) was conducted [37]. The studies described in [38,39] analyzed the application of software-defined network (SDN) issues for the aerial communication network (consisting of airplanes, balloons, and airships) for user-to-user communication with a focus on backhaul connectivity. The implementation of the architecture in a software-defined UAV network that uses multiple radio technologies was proposed. The study stated that UAVs are used in different organizations and for academic issues for the purpose of increasing wireless network performance for mobile users [39]. 

The authors in [40] focused on the improvement of dense network capacity performance and proposed a caching-based mechanism using UAVs to use together with other BSs. Mainly, the study considered the storage of content and the delivery to users following increased demand. The main concern was that the positioning of UAVs would affect network performance metrics, such as throughput, coverage, and connectivity. The objective was to address the mismatch between the wireless communication capacity and the network traffic caused by user requests by focusing on multiple UAV-assisted networks to improve the performance by using terrestrial heterogeneous networks [41]. The study was planned to evaluate and analyze the positioning of UAV-BSs to improve user coverage and throughput. The study described in [42] used a methodology to optimize the trajectory of a UAV to offload traffic at the edge regions of three adjacent BSs. The proposed iterative algorithm alternatively optimizes user scheduling. The study described in [43] focused on the wireless network system using a UAV-assisted network or BSs as an access link between ground users. The study proposed an algorithm for network performance optimization of A2G with consideration of the location angle of the UAV. In [44], a proposed gradient-based chaining controller algorithm was applied as the mobility control algorithm to achieve optimal positioning of multiple UAV communication. The purpose of this method was to estimate the performance of near-to-near UAV communication links. The studies described in [45] carried out 3D positioning of UAV-BSs for future 5G wireless networks. These studies were initiated to maximize user coverage and network performance [46]. In the study described in [35], an approximation algorithm was proposed for positioning UAVs to maximize user throughput. The approximation algorithm was applied to compute the position of each UAV to the overall system user throughput; this maximized the data rate demand and path of each flow. The paper contributed to the formulation of the UAV positioning problem for the optimization of user throughput based on traffic demand. The SDN controller, which maintains the traffic demand information for all links and flows, has been used to predict the current UAV positions and associated users [3]. However, the previous literature has rarely studied the capacity of UAV support for data collection networks. As Table 1 shows, we compared some previous works with our study based on their objectives, methodologies, and algorithms used, as well as their results.

Thus, we focused on the evaluation of different performance measures that are suitable to quickly [30] predict class and grid-based UAV positioning through a large number of real datasets. In this study, we focused on class and grid-based data collection with several mobile users and different numbers of UAVs. We based this work on unique features from other previous works, such as using a real data collection environment rather than simulations and applying ML-DL approaches, which are preferable for real and complex environments. DL approaches have the advantage of representing and extracting the most representative features from multi-sourced datasets. 

## 3. Data Collection and System Model

This section describes the data collection methods, such as experimental setup, data collection procedures, the hybrid architecture of MLP-LSTM for the proposed system, and system model structure, the main parameters used to build our proposed system. 

### 3.1. Experimental Setup

**Figure 2:**
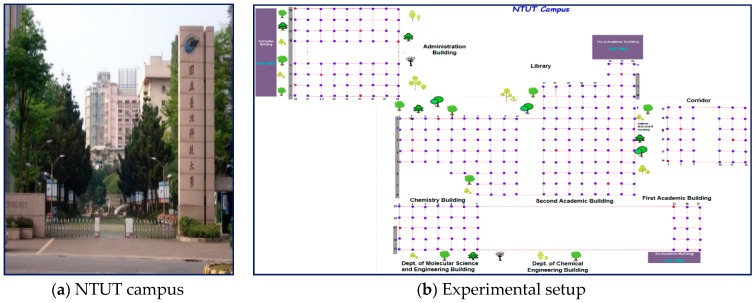
Real structure of a working environment.

According to the rules and regulations of Taiwan UAV flights, we assumed that altitudes higher than 120 are not used, as their use is prohibited around airports or in areas where aircrafts are operating; and only flights in good weather conditions and daylight hours are undertaken; and in sensitive areas, including government or military facilities, flights should be considered on the plan of data collections. Therefore, with the consideration of these general rules and regulations, we designed our data acquisition procedures and environment as follows. For this work, experimental data was collected by real-time recording from the real environment at the National Taipei University of Technology (NTUT), Taiwan. We collected OFDM signals from the temporally designed UAV-BSs, including the SNR of each BS. Figure 2 depicts an outline of the working environment covering 500 m × 300 m and the square grid points. The real working environment contained both LoS and NLoS locations, because the setup was enclosed by towers, trees, and other shadowing effects, as shown in Figure 2a. The target area was partitioned into grids to collect the OFDM signal dataset. Mostly, the outdoor environment was divided into 1 m × 1 m and 2 m × 2 m squared grids to construct a radio map. Reducing the size of grid points improved the positioning accuracy, but it increased the site survey costs in a scalable environment. In this study, the working set-up was divided into small sub regions using 2 m × 2 m square grids to collect the signal values via smartphones to build a radio-map. The received signal values were scanned using a war-sensing technique in each reference point (RP) from reachable APs and temporarily deployed UAV-BSs, and then the RSS values were stored in a central server (PC).

Figure 2b illustrates the experimental set-up of the open outdoor area where the real received signal data measurements were taken in each grid point. In this figure, the blue dots represent the training reference points (722 RPs) and the red dots indicate the testing reference points (85 RPs). The purple color indicates the locations of buildings that act as temporarily deployed UAV-BSs to collect the OFDM signal values as cellular data. In order to train and measure the performance of the proposed system, we split the data source into a training set and testing set. A total of 85 reference locations were selected randomly for testing purposes to assess the system accuracy. Table 2 presents a summary of the training and testing data sources. The next section contains an explanation of the data collection procedures.

### 3.2. Data Collection Procedures

To evaluate the user throughput transmission rates, we conducted an experiment involving data measurement with 900 MHz bands from the National Technology University of Technology (NTUT) in Taiwan. To collect our data, UAVs were used, as a Tx was placed at three buildings (shown on Figure 2b). The data measurements were performed at both Tx-Rx sides using the Tx-Rx BS, mobile, and user equipment (UE) features. In accordance with [5], the location pair of horizontal and vertical (elevation) angles was enabled to specify and estimate the direction of the maximum received throughput and power. In particular, we used the K-means algorithm to cluster the collected datasets into three classes (class 1, class 2, and class 3) based on their signal strength values and their LoS/NLoS from the Tx. In each class, squared grid areas were used for OFDM signal data collection. 

**Figure 3:**
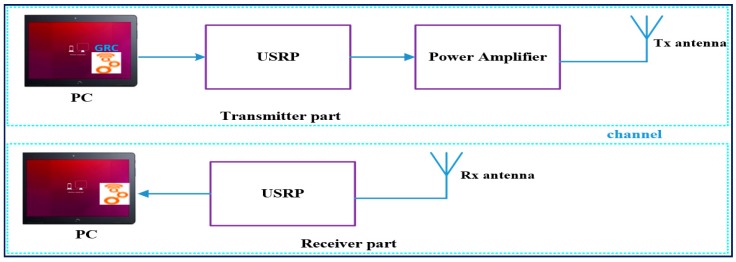
Collecting orthogonal frequency-division multiplexing (OFDM) signal values from UAV-base stations (BSs).

We used three different smartphones—Samsung, Huawei, and Apple devices—to reduce the signal variation due to device diversity and also to reduce the dataset collection time. At each grid point, 35 RSS values were collected periodically in 3 seconds on three different days to mitigate signal fluctuations due to environmental changes. The UAV-BSs were placed at the top of each building—the complex building, third academic building, and sixth academic building—and the distance between buildings was around 400 m. Therefore, we measured signals from UAV-BS heights of 40, 50, and 60 m to the ground user in order to adapt the environment to replicate a real scenario. As shown in Figure 3, to measure the real OFDM signal to each UAV-BS, we used dipole antennas at the Tx and Rx, a Universal Software Radio Peripheral (USRP) system, a power amplifier, and computers. At the transmitter side, the USRP device was used to build the software defined radio (SDR) system to generate the real signal from the Ubuntu loaded computer. However, the power of USRP is very limited, so we used the power amplifier to increase the signal power. Besides, we used dipole antennas at both Tx and Rx, and the output power of the transmitter antenna was set at 29.5 dBm. On the receiver side, the computer read and stored the OFDM signal values from the received signal through the USRP device at each grid point. However, on the Rx side, the USRP did not need the power amplifier just to receive data from the receiver antenna. The GNU Radio Companion (GRC) source code written in C language and running on the Ubuntu platform was used to implement software defined radios. The total bandwidth for each UAV-BS was 3.2 MHz, and the carrier frequency was 900 MHz. When the UAV-BS height increased and there was a LoS, the strength of the received signal value decreased at each reference point. However, if many buildings and trees were available when the UAV-BS height increased, the transmitter was able to transmit better signal values than the UAV-BSs with the smallest heights. 

The A2G access link heights of UAVs were differentiated based on the values of 40 m (UAV_1_), 50 m (UAV_2_), and 60 m (UAV_3_). The location of each UAV was estimated by its longitude and latitude values. UAV_1_ had a longitude of 121.53598 and a latitude of 25.042971; UAV_2_ had a longitude of 121.53554 and a latitude of 25.043390; and UAV_3_ had a longitude of 121.53465 and a latitude of 25.0433980.

Each UAV-BS reached each of the specified classes, and we used RSS and SNR directly from UAVs. We also used the central distance location for UAVs to estimate the UAV and user’s locations as a longitude value of 121.535710 and a latitude of 25.043095, which enabled us to compute the distance between UAVs and ground user locations. We collected a total dataset record with 25,023 values. For system simulations and evaluations, we used the main system parameters, as indicated in Table 3. 

### 3.3. The Hybrid of MLP-LSTM Architecture for the Proposed System

**Figure 4:**
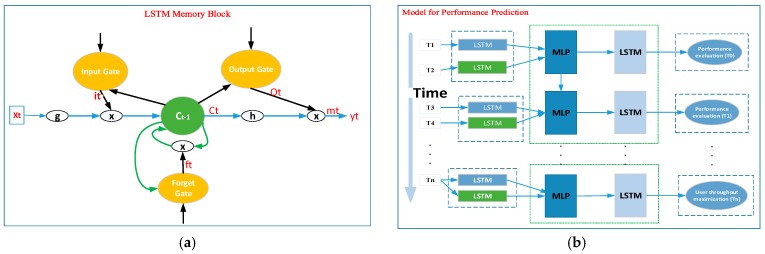
(**a**) LSTM memory structure; (**b**) structure of our prediction model.

There are many DL architectures, including convolutional neural networks (CNNs) and RNN. Feed-forward neural networks, such as CNN, do not have cyclical connections, and they do not have input memory. On the contrary, the recurrent connections and memory of RNN can recall the whole history of prior inputs to each output [48]. However, RNN has the problem that the gradient is small when RNN is the learning gradient in the process of backpropagation through the activation function. In particular, deeper hidden layers have a worse problem. In order to handle this issue, certain RNN structures, such as the LSTM, have been designed to give the memory cells the ability to control when to forget certain information [49]. So, LSTM models are becoming progressively familiar [50]. This study adopted a basic LSTM neural network. A diagram of the memory block is shown in Figure 4. For our study, we aimed to jointly model MLP with LSTM-based algorithms for use in a dataset with a large number of OFDM signals classified into three classes from a specific data collection area. The amount of training data was large, the classes and altitudes of UAV scales (such as classes 1, 2, and 3, and 40 m, 50 m, and 60 m) varied, and the single layer LSTM had difficulty modeling them. Thus, the training sequences varied at different time scales and needed to be processed by multiple nonlinear hidden layers. Recently, using Google’s acoustic modeling system, it has been demonstrated that the multiple layer LSTM allows the network to learn at different time scales over the input [51]. The integration of MLP-LSTM can control or reduce the vanishing gradient and backpropagation problem which may be faced on MLP approaches. Therefore, we chose to use the MLP-LSTM learning architecture to build the whole system. Moreover, the user location and UAV altitude from the ground are shared important information and they are highly correlated with each other. Thus, we proposed the use of a multi-layer based MLP-LSTM based learning architecture to jointly learn the training and prediction model. The key concept of multi-task learning [52] is to learn several tasks simultaneously with the aim of gaining mutual benefits; thus, learning performance can be improved through parallel learning while using a shared representation. Therefore, it was reasonable to expect better results from our application through this learning framework. Another advantage of this learning architecture is that a single data source can be used and representations can be shared.

As shown in Figure 4, the STM memory architecture is able to use time series with long time spans and automatically determine the optimal time lags for prediction. In this study, we chose to use the LSTM to model the long-term temporal dependency of user mobility and to estimate UAV positioning. The LSTM network computes a mapping from an input sequence, x = (x_1_…x_t_), to an output sequence, y = (y_1_…y_t_), by calculating the network unit activations using the following equations iteratively from t = 1 to t:

*Block input: g* (*W_z_x^t^* + *R_z_y*^*t*−1^ + *b_z_*), *Input gate: i_t_* = σ (*W_ix_x_t_* + *W_im_m*_*t*−1_ + *Wi_c_c*_*t*−1_ + *b_i_*), *Forget gate: f_t_* = σ (*W_fx_x_t_* + *W_fm_m*_*t*−1_ + *Wf_c_c*_*t*−1_ + *b_f_*), *Cell state: c_t_* = *f_t_* ⊙ *c*_*t*−1_ + *i_t_* ⊙ *g* (*Wc_x_x_t_* + *W_cm_m*_*t*−1_ + *b_c_*), *Output gate*: o_t_ = σ (*Wo_x_x_t_* + *Wo_m_*m_*t*−1_ + *Wo_c_c_t_* + *b_o_*), m_t_ = o*_t_* ⊙ h(c*_t_*), *Block output*: y*_t_* = ∅ (W_y*m*_ m*_t_* + b*_y_*), (1)

where *i*, *f*, and *O* represent the input gate, forget gate, and output gate; *c* and *m* are the activation vectors for each cell and memory block; and the weight matrices, *W*, and bias vectors, *b*, are utilized to build connections between the input layer, output layer, and memory block. Here, ⊙ represents the scalar product of two vectors, σ(.) denotes the standard logistics sigmoid function, and g(.) and h(.) are the cell input and cell output activation functions and generally centered logistics sigmoid function. Figure 4a,b, show the basic structure of the LSTM memory block, the LSTM memory structure, and the structure of our prediction model. The LSTM update gate is the combination of the input and forget gates, and the previously hidden state, *h*, is directly connected to the reset gate [41], whereas in LSTM, the memory content to be used or seen by other units/cells in the network is managed by the output gate, similar to the LSTM cell, the hidden state of time, *t* − 1, and the input time series value, *x*, at time *t*. Figure 4b, depicts the combination prediction structure of MLP-LSTM for the construction and prediction of our system model. As shown in the figure, performance evaluation (i.e., UAV-positioning and throughput maximization) was used as the target output for our system with the input and hidden layers of the user and UAV variety datasets determined by combining MLP-LSTM structures.

### 3.4. System Model

**Figure 5:**
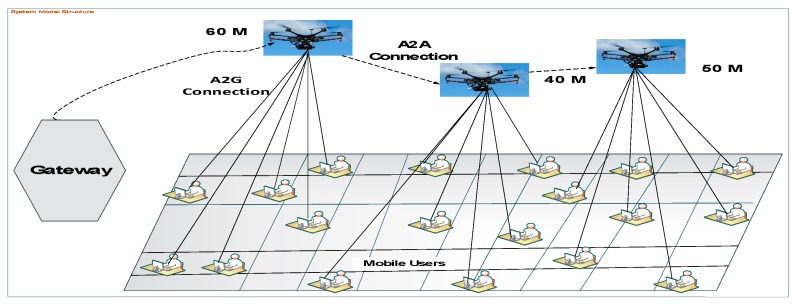
Illustration of the UAV-enabled system model.

In this section, the flow of information and power structure from T_X_ (UAV), represented as *M*, to R_x_ (mobile users), represented as *U*, with different altitudes and distances are elaborated. Figure 5 represents the sample structure of A2G and A2A access link communication for data transmission and user handling services. Our system model layout contains the network environment with three *M*_s_ as the BS, and 35 *U*_s_, represented by U_1_, U_2_, U_3_, …, U_n_, which are serviced by the deployed M. The figure focuses on the A2G link of M-U communications and further assumes that the A2A channel access link and all users operate over the same frequency band. In addition, all of the ground U are assumed to have received OFDM-RSS and SNR values simultaneously. For the A2A channel link access, the network communicates with the gateway node. Based on the system model structure, all *M*s communicate with each other and are positioned with different altitudes: M_1_ as 40 m, M_2_ as 50 m, and M_3_ as 60 m. 

Consider *G* (*M*, *E*) for the channel communication network, where *M* is the set of UAVs plus the communication node of a gateway (GW) that connects the UAV network to the core network and internet. For the purpose of positioning issues, we used *M* as a BS whose position cannot be changed and *E* as the set of links between the *M* or the *M* and the gateway. In the diagram, *M_s_* to *G* denotes the set of connected components of *G*. A link, *L* (M_1_, M_2_, and M_3_), exists between any two *M*_s_ (M_1_, M_2_, and M_3_) if and only if they are within the transmission range of each other, i.e., L_M1_, L_M2_, L_M3_ ∈ E > distance (P_M1_, P_M2_, P_M3_) ≤ T_x_ R_M2M_, where T_x_ R_M2M_ is the transmission range for A2A communication, and P_M1_, P_M2_, and P_M3_ denote the positions of M_s_, M_1_, M_2_, and M_3_, respectively. Each *M u*∈*U* provides a communication service to a set of users, *S*(*u*), where each user is receiving OFDM signals from a single *M*. Assume that each *M* would communicate with other *M*_s_ concurrently without interfering with the communication of the other M_s_. All three M_s_ have the same transmission range, TxR_M2M_ for A2A access links and TxR_M2U_ for the A2G access link. The transmission range for the A2A access link is larger than that for A2G, i.e., TxR_M2M_ > TxR_M2u_. The channel used by an *M* for communication with its associated users is different from the channels used by all its neighboring *M*_s_, i.e., if *C_M_* denotes the channel used by an *M_n_* to communicate with its associated users, then *C_n_* = *C_n_*_1_, *∀_n_*_1_∈*N*(*n*)*, ∀n*∈*n*, where *N*(*n*) represents the set of neighbors of *M*. This effectively eliminates the interference between users associated with neighboring *M*_s_. 

For the simulation and evaluation of our system, we mainly used the following main system parameters, as indicated in Table 3. Then, we computed the values of RSS distribution and user throughput at each UAV distance, height, and elevation angle for the purpose of estimating the positions of UAVs for user throughput maximization.

## 4. Proposed System Description

**Figure 6:**
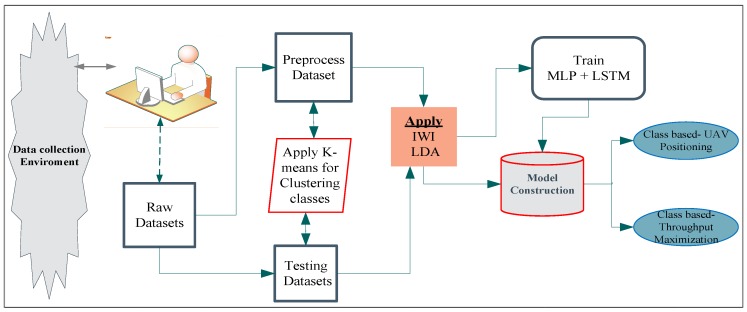
Proposed system architecture.

Figure 6 is a detailed diagram of our proposed system. The proposed system architecture contained a data collection environment (which is classified with grid points). After the identification of our data collection areas and grid points, the mobile user gathered their required OFDM signals, which contained parameters, such as the index number, RSS, received power strength, and SNR. Then, the data preprocessing task was conducted. After the dataset had been cleaned and arranged with an appropriate format (i.e., Excel or CSV), the training phase was performed. Initially, users were randomly distributed on the ground and received OFDM signals from the three UAVs located on the tops of the three buildings. The data collection environment contained the real characteristics of trees, buildings, and flow of users. Users were located at zero altitude, and the collected dataset was delivered to the next phases of preprocessing and online dataset for the training and testing tasks. After the required dataset had been collected, preprocessing was conducted to avoid unwanted and misfiled datasets. To preprocess the dataset, we focused on important values or parameters, such as the RSS, distance, throughput and elevation angle, and the grid number for user locations. To perform these phases, an interpolation method was used to calculate or approximate user-to-user distances. Iterative weighted interpolation (IWI) methods and LDA were used for the execution of dimensionality reduction, because this enabled the structure and size of the raw datasets to be reduced to improve the time and space complexity.

In the training phase, the training (offline) datasets were used based on the inserted parameters and their values. Simultaneously with the preprocessing phase, the K-means algorithm is useful for clustering to obtain the groups of objects that have common characteristics in large datasets based on the dividing technique formed on the centroid. The K-means algorithm produces better results for clustering than other algorithms [33]. Consequently, in our system, the K-means algorithm was used for the automatic clustering of classes from the data collection environment through their signal similarities and power value estimations. We also used the geographical layout, the location of the environment, and the signal strength differences to classify data into three classes. To construct the appropriate model, we attentively trained the MLP and LSTM for classification and regression tasks to achieve user throughput maximization and evaluation on the classes and grid point. 

In terms of testing phases, the online dataset also contributes to the model construction phase. So, the model was constructed through a combination of training and testing phases. As depicted in the figure, based on the constructed model, the final expected outcome of our work was to estimate, predict, and analyze the values of user throughput separately as common user throughput maximization values and to use them for UAV positioning based on the better-identified user locations. After the design of our proposed system, the following sections describe scenarios’ where UAV positioning can be applied for user throughput maximization, such as original datasets, reduced features, and the estimated values of user throughput at each grid point, and the reduced feature datasets collected on different days and grid points achieved maximum throughput.

The proposed algorithms were implemented through the Python 3.7 programming tool, which is a powerful multi-purpose programming language with a TensorFlow framework in conjunction with multiple python packages and libraries, like keras and sklearn [47], because of their advanced and helper functions that make it much easier and faster to write DL algorithms, due to the familiarity of the researchers with the tool. The simulation was run on a PC with Intel Core i7-4790 3.60 GHz CPU and 20.0 GB RAM.

Case 1: Ensuring all Associated Users Remain Inside the Transmission Range

If the exact UAV position estimated by the DL approaches is inside the working areas, then it is guaranteed that all users will be within the transmission range of the UAV. However, if it (the wanted user location) is outside the coverage of UAVs, then some users might be outside the transmission range. After collecting the required datasets, we applied the data cleaning technique [32] to control unfilled and out of range datasets, as indicated in Equation (2). This technique helps to remove abnormal data, which causes the machine to learn wrongly. Hence, this technique helps to ensure that the proposed approach easily learns from the collected datasets. Therefore, we used *D* to represent the outside range of the datasets, where D is given as:

(2) $$D\in AvgRss-3\sqrt{\frac{1}{N}\sum_{i=1}^{N}{\left(X_{i}-AvgRss\right)}^{2}}<X_{i}<AvgRss+3\sqrt{\frac{1}{N}\sum_{i=1}^{N}{\left(X_{i}-AvgRss\right)}^{2}},$$

where ($X_{1}$, ${\;X}_{2}$, …, $X_{N}$) represent the RSS of mobile users, and AvgRSS is the average of the total users’ RSS values.

For simplicity, we applied a clustering approach based on the K-means algorithm to generate classes easily. The PL model of LoS and NLoS, as shown in Equation (3), was used, where *d* is the distance between the UAV and the user, *α* is the path loss exponent, *β* is the average channel gain at a reference point, *f* is the carrier frequency, ξ models the shadowing effect, which is a Gaussian random variable, *N*(0, $\sigma_{S}^{2}$), in the states as *S* = LoS or *S* = NLoS, and the mean PL is computed as:

(3) $$PL_{S}\left(d\right)=\alpha_{S}10\log d+\beta_{S}+20\log f+\xi_{S}.$$

The RSS values collected from the three different UAV heights and the distance estimation from UAVs to mobile users were assessed for each LoS and NLoS using Equation (3) through the interpolation method, i.e., using sample points to estimate values at other unknown points to predict unknown values [35]. The parameters were estimated using least-square errors, as shown in Equation (4). The estimated parameters contain $\left(\alpha_{S},\beta_{S},\sigma_{S}\right)$. The least-square method was used for parameter estimation, since the technique is simpler and less complex:

(4) $$\underset{d}{\min}\sum_{i=1}^{N}{\left\|{\hat{PL}}_{S}\left(d\right)-PL_{S}\left(d\right)\right\|}^{2}.$$

To analyze and predict the 3D wireless user throughput, we effectively provided UAV-BSs to accurately evaluate the A2G links of mobile users, including the user’s current communication status as LoS/NLoS, the distance, the user’s signal strength, and the path loss in the same scenario. To determine the vertical distance between UAV and mobile users, Equation (5) was used, where U is user {U…U_35_}, and each UAV is represented by *M* located at the given heights of 40, 50, and 60 m. To compute the horizontal distance Equation (6) and elevation angle, θ, using Equation (7), the angle variation and user in the area were computed:

(5) $$d_{um}=\sqrt{{\left(h^{2}-r_{um}\right)}^{2}},$$

(6) $$r_{um}=\sqrt{{\left(x_{u}-x_{m}\right)}^{2}+{\left(y_{u}-y_{m}\right)}^{2}},$$

(7) $$\theta u_{m}=tan^{-1}\left(\frac{d_{um}}{r_{um}}\right)$$

where *h* is the altitude of the UAV, $r_{um}$ is the horizontal distance between *M* and *U*, and ($x_{u},\;x_{m}$, $y_{u},\;y_{m}$) represents the locations of *U* and *M* in the horizontal dimension. These helped to determine the number of users being served by a UAV in given class. The A2A distance was computed as:

(8) $${\mathrm{UAV}}_{m,n}=\sqrt{\left(m_{1}-m_{n}\right)}{)}^{2},\;m,\;n\in M,\;m\neq n.$$

Next, we considered the wireless transmission over the A2G access links from the UAVs to the ground users as well as over the A2A backhaul links among the UAVs. For both A2G and A2A links, we supposed that the wireless channels were dominated by the LoS communication link. Therefore, we used the free space path loss model (Equation (9)), as commonly used for the UAV communication links in previous works [8,20,21]. Therefore, the channel power gain from UAV_1_ M_1_ to user U_n_ was expressed as:

(9) $$h_{m,U}\left(u_{m}\right)=\beta_{0}d^{-2}{}_{m,U}=\frac{\beta_{0}}{H^{2}+{\left(U_{m}-W_{u}\right)}^{2}}\;,\;m\;\in M,\;u\in U.$$

However, due to the high LoS channels over both A2A and A2G links, the co-channel transmission may lead to severe interference among different users and UAVs. To solve this issue, we considered orthogonal transmission among different A2A and A2G links to avoid any co-channel interference, in which each link was allocated a dedicated frequency band.

Case 2: Estimate the Values of User Throughput for Each User at a GridPoints

For the purpose of serving all users within the transmission range, UAVs should contain all the users in the working grid points. To satisfy this issue, the user throughput values should be estimated at the specified grid points within the working environment. Therefore, we proposed a positioning algorithm, iterative weighted interpolation positioning (IWIP), to evaluate and balance user throughput values to establish a fair distribution of signals to all the users. Then, the evaluation was done to select the grid point that contains the maximum estimated user throughput values. Based on the generated results of the estimated values, all users were served in the Tx and Rx of the UAV until the throughput was maximized using the UAV position, user location, and distance. In short, the tasks of positioning can be expressed as follows:

(1) After determining the data collection area, identify a grid of points (user location points given service by UAV) in the working area.
(2) Compute the desired UAV positions that are required to serve all users based on the number of users and their positions.
(3) Adjust the desired positions of UAVs so that all Rx are inside the Tx range.
(4) The newly adjusted UAV positions should be contained within the whole working line of grid points and outside the working area.
(5) Estimate or sum up the total values of the user throughput at each grid point.
(6) Move the UAV to the newly adjusted locations to handle all users within the Tx range.

**Figure 7:**
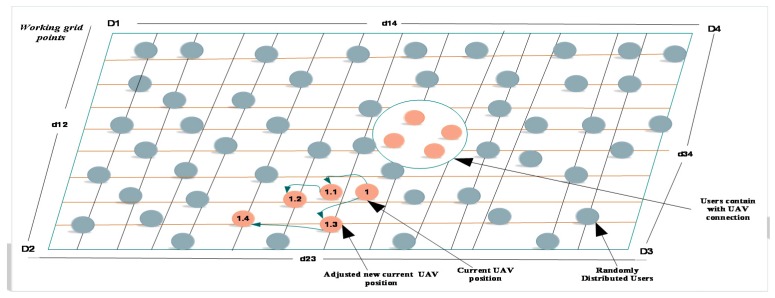
The principles of iterative weighted interpolation positioning.

Finally, the grid points were re-evaluated, and the grid points with the maximum estimated user throughput were selected as the newly positioning area. According to [47], in order to estimate the user throughput for users at a specified grid point, estimate the transmission rate for each user needs to be estimated. Figure 7, depicts the methods of IWIP and the whole structure of grid points inside our working area. All the circles and ash grey colored lines illustrate the probable locations of mobile users, coverage, and the remaining grid points. For instance, the atomic tangerine colored grid points inside the circle show better estimated user throughput values than the other points. The remaining or separate atomic tangerine colored grid points show the estimations of user throughput values. For example, 1 is the current location of a UAV, and the next points, 1.1, 1.2, 1.3, and 1.4, are considered the newly adjusted UAV positions. At these points, the UAV compares the user throughput values and decides to allocate the next points based on their data rate demand, because the principle of our proposed algorithm tries to fairly assign the user throughput values for all users. The symbols represented as D1, D2, D3, and D4 indicate the whole area and user-to-user distance measurement for all grid points in each class.

Case 3: Finding the Grid Points Achieved Maximum and Total Throughput Maximization

The points with the total and maximum estimated user throughput were determined to compute the user throughput capacity for each user at a grid point, where the user throughput value at the current grid point was greater than the current maximum value. Then, the point with the estimated maximum throughput, *P_max_*, was determined. After identifying the highest sum values of user throughput for all users, they were estimated as R_sum_ (T) = $\sum_{u=1}^{U}\mathrm{Ru}\;\left(T\right)$, which is a combination of the time allocation, *t*, for A2G. Therefore, it guaranteed an equal throughput allocation to all users and maximizes their sum-throughput. This can be described as follows:

▪ The algorithm iterates over all the grid points inside the working area until it gets the *T_max_* (maximum throughput value) and *P_max_* (maximum user throughput position).
▪ The optimal UAV position is considered, i.e., d_um_ (P_u_, C_cont_) ≤ R_cont_, which enforces that the UAV should remain inside the wanted area while the optimal position is found.
▪ To maximize (UAV position) $\sum_{j\;\in \;S}\mathrm{tj}\;\mathrm{Cj}$ ct T: d_u_ = distance (P_u_, P_n_), ∀n ∈S, the distance between each user and the UAV is computed; C_u_ = f(P_t_, d_u_, I_u_), ∀_j_ ∈S shows that the throughput capacity is a function of the transmit power, the distance between the user and UAV, and the interference experienced by the user. Where *C_t_* denotes the common user throughput and *d* is a feasible set of *T*, and *I* and *P* are the interference and power transmission, respectively.

### Principle of Iterative Weighted Interpolation Positioning (IWIP)

In order to measure the U2U distance, we proposed an IWIP scheme based on signal differences or variations. This method can easily achieve multi-user localization with higher positioning accuracy, because it combines the advantages of the centralized positioning scheme and decentralized positioning scheme [32]. In accordance with [52], we applied the IWIP because mobile users can use it to search and collect several OFDM-RSS values at a time and it is a better way to estimate the positions of UAVs. In addition, it is important to reduce the power consumption compared with the centralized positioning method. As shown in Figure 7, we assumed there were mobile users at positions of {U_1_...U_n_} with the position of *P_u_* in between U_1_ and U_n_, and we set the ratio, $\frac{d_{U1\mathrm{Un}}}{D_{u1\mathrm{un}}}=\frac{d_{u1}}{d_{u2}}=\mathrm{du}1\mathrm{un}$. Therefore, we computed P_u_ as:

(10) $$p_{u1un}=u_{1}+\frac{d_{u1un}}{d_{u1un}+1}\cdot u_{1}u_{n},$$

where $p_{u1\mathrm{un}}$ is a vector from $p_{u1}{\;\mathrm{to}\;p}_{\mathrm{un}}\;\left(p_{u1\mathrm{un}}=p_{u1}-p_{\mathrm{un}}\right)$. After computing all the positions of users, and to compute the locations of users and UAVs, the IWIP was applied after taking the weighted interpolation of the positions of {U_1_…U_35_}. Then, the two weighting factors that represent the weighted location were computed:

(11) $$E_{pu}=\frac{p_{un}p_{u1un}+p_{un}p_{u1un}}{p_{un}+p_{un}}=w_{u1un}P_{U1un}+w_{un}p_{u1n},$$

where E_pu_, is the estimated position for mobile users. In general, our proposed algorithm was applied to identify and cluster the classes in the working area and to estimate the values of grid points to compute and predict the total user throughput at each class, based on the preferred UAV height and distance, and to assess the overall performance of our MLP-LSTM model in terms of class and grid point based analysis of user throughput values. Finally, to test our model for positioning and user throughput maximization accuracy, we used the root mean square error (RMSE), mean square error (MSE), and mean absolute percentage error (MAPE) performance evaluation methods and system performance metrics with different scenarios. They were computed as follows:

(12) $$\mathrm{RMSE}=\sqrt{\frac{\sum_{i=1}^{n}{\left(y_{i}-y\right)}^{2}}{n}},$$

(13) $$\mathrm{MSE}=\frac{1}{n}\sum_{i=1}^{n}{\left(y_{i}-y\right)}^{2},$$

(14) $$\mathrm{MAPE}=\frac{1}{n}\sum_{i=1}^{n}|y_{i}-y|,$$

where n is the total number of predicted values, y_i_ is the actual value, and y is the predicted value. The MSE of the distance estimate was proportional to the user-to-user distance.

## 5. Discussion of the Experiment and Results

In this section, we describe simulations using both classification and regression schemes to analyze the performance of the proposed algorithm. The simulation was done using real data collected from an outdoor environment. Different parameters, such as the number of epochs, hidden layers, hidden units, types of optimization, and the activation function, were adjusted and used as part of the experimentation to train the MLP and LSTM models. In the classification scheme, we compared MLP-LSTM performances with SVM, LSTM, and MLP algorithms in different scenarios; more specifically, we used original datasets (scenario 1), used reduced features only and estimated the values of user throughput for each user at each grid point (scenario 2), and used reduced data collected on different days/times and found the grid points in which users achieved maximum and total throughput (scenario 3). In the regression scheme, testing was performed in similar scenarios to prove the effectiveness of the proposed approach. In all scenarios, we considered the total user throughput analysis based on the positions of UAV with altitudes of 40, 50, and 60 m within three classes. 

**Figure 8:**
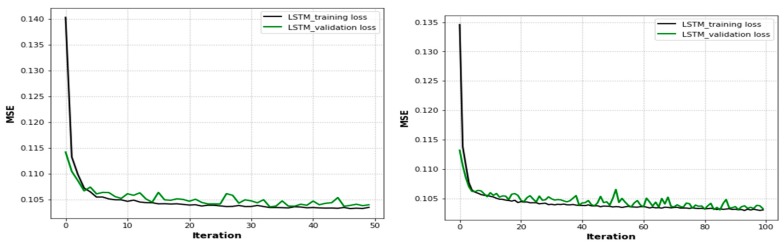
LSTM-based loss function plots.

**Figure 9:**
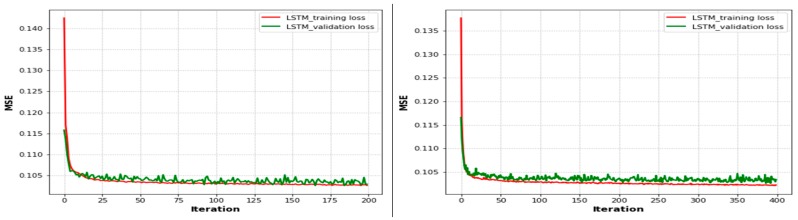
LSTM-based loss function plots with different epochs.

**Figure 10:**
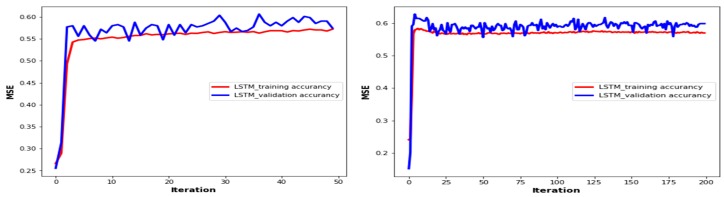
LSTM-based training and testing accuracy plots.

When the UAVs were positioned at their optimal locations, the time passed and the number of epochs increased the user throughput in the training and testing phases, as shown in Figure 8, Figure 9 and Figure 10. Therefore, in Figure 8, we compare epoch 50 (training and testing losses overlapped after 0.114 to epoch 8 and were then parallel until the end of the iterations) and epoch 100 (the training and testing losses overlapped after 0.113 to epoch 6 and were then parallel until the end of the iterations) based on the LSTM approach. After the performance degradation exceeded some threshold, the process of optimal UAV positioning had to be repeated. In both scenarios, as the user throughput dropped, the optimal positions for UAVs were recomputed again based on the existing conditions, and the UAVs were moved to them. For user mobility, as one would expect with higher user speeds, the throughput dropped quickly. In these results, the time it takes for a UAV to move from its current position to the optimal position was ignored. Finally, as shown in both figures, the training loss decreased as the number of epochs increased. The training loss converged quickly to a low value, since the error on the testing set closely matches the training set. The network did not over-fit the training data. 

Figure 9 compares epochs 200 and 400. The training loss converged quickly to low values of 0.115 and 0.116, respectively, after approximately 15 epochs based on the performance of the LSTM approach with training and testing phases. As shown in the figure, the training loss decreased when the number of epochs increased. After the performance degradation exceeded some threshold, the process of optimal UAV positioning had to be repeated. Since the error on the testing set closely matches that of the training set, the network did not over-fit the training data. Moreover, we used different activation functions, such as sigmoid, rectified linear unit (ReLU), softmax, and tanh, in order to squash the output of the neural network to be within certain bounds. Then, we performed many experiments to find the optimal values of parameters to obtain less MSE and a short training time. Therefore, after some experimentation, we selected the parameters and trained the LSTM model using a batch size of 1500, with varied iterations of 50, 100, 200, and 400 epochs, and 512 hidden units. We compiled the network using the Adam optimization algorithm and the tanh activation function. The loss metrics used for evaluating the prediction performance of the model and optimizing the weights were chosen to be the preferable MSE.

Figure 10 shows the accuracy of the training and validation phases with the number of epochs based on the LSTM approach. As shown in the figure, the training loss decreased with an increasing number of epochs. The training accuracy converged quickly to low values of 0.55 and 0.60 for 50 and 200 epochs, respectively. However, the validation accuracy also converged at around 0.53 and 0.57 for 50 and 200 epochs, respectively. As a result, the error on the validation set closely matches the training set and loss plots, and the effect of input parameters on the number of iterations during the training of the LSTM decreased when the number of iterations increased. However, the model indicates it is capable of accurately measuring the user throughput values because the training accuracy and validation accuracy sets are closely matched based on their iterations and the model did not have an overfitting problem.

**Figure 11:**
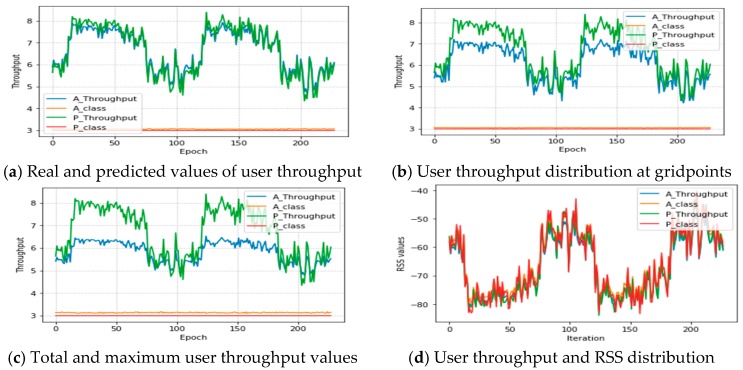
MLP-LSTM—actual and predicted values of user throughput.

Figure 11 shows the actual and predicted user throughput values as well as the predicted and actual class values with the number of epochs and different conditions. As illustrated in the figure, both the actual and predicted values for the user throughput and class values are approaching or are plotted similarly to the increment of epoch values. This indicates that the proposed algorithm can easily adapt and make efficient predictions with the actual values. For instance, Figure 11a shows the original dataset (scenario 1), in which the actual and predicted throughput values are plotted similarly; Figure 11b indicates the performance of scenario 2, in which the actual and predicted values are compared after using LDA, and the predicted values have excellent better estimation; and Figure 11c indicates the performance of scenario 3 in which the actual and predicted values are compared. A promising result is shown for the prediction task for all grid points. Finally, Figure 11d shows the comparison of RSS and the user throughput values. The proposed algorithm estimated user throughput distribution for all the classes and grid points with a similar distribution of signals.

**Figure 12:**
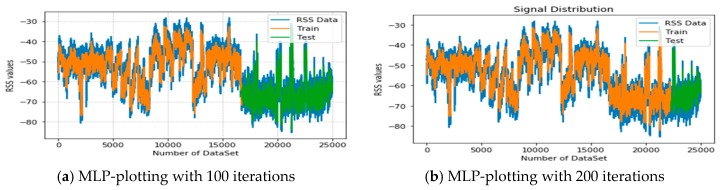
Illustration of the MLP-based signal distribution in each class

In Figure 12a,b, the estimation of RSS signal distribution with the incremental number of dataset values is shown based on the MLP approach. In the figure, blue shows the distribution of the OFDM signal, and yellow with blue and green with blue show the datasets classified for training and testing (validation), respectively. Figure a has 100 iterations and the second figure has 200 iterations. In both figures, the maximum result reached RSS values of −30 to −90. Finally, the result indicates that the real signal distribution and the predicted values are plotted similarly. This indicates that the MLP algorithm also showed a better performance, which can classify the distribution of RSS signals over different iterations based on the elevation angle, UAV altitudes, and distance.

Table 4 shows the user throughput performance in the classification scheme under different scenarios. The UAV positioning accuracies for scenario 1, scenario 2, and scenario 3 were 80.20%, 87.23%, 94.11%, and 96.73%; 87.00%, 92.03%, 95.14%, and 98.33%; and 89.1%, 96.12%, 96.21%, and 99.53%, respectively, using the SVM, MLP, LSTM, and MLP-LSTM based classifiers. The performance accuracy of all classifiers was greatest in scenario 3 since the LDA resolved outliers and removed noise to allow accurate positioning. In scenario 3, the accuracy levels of MLP and LSTM were highly improved, and the values approached each other. The hybrid of MLP-LSTM gave accuracies of 96.73%, 98.33%, and 99.53%, respectively, in each scenario. Thus, the UAV positioning and user location improved based on their user throughput values in each class and grid points. The performance of LSTM was also better than the others, while it was lower than that of MLP-LSTM. The reason for this is due to the higher learning capabilities in a real and dynamic environment. Moreover, MLP-LSTM has better UAV positioning accuracy than MLP and SVM, because MLP-LSTM combines the highest hidden and memory capacities in a large dataset structure. 

**Figure 13:**
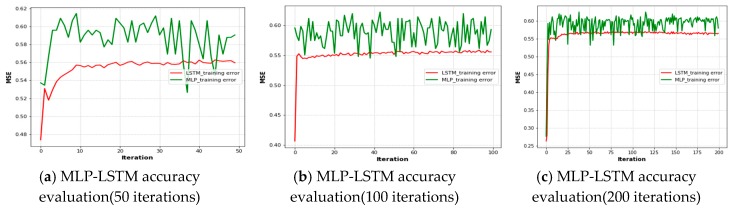
The effects of user mobility based on UAV altitudes in the grid points based on the MLP-LSTM model.

Therefore, the proposed approach has better positioning accuracy, as it integrates MLP with LSTM, and the selected grid points have maximum user throughput, so the model is more flexible with respect to complex and dynamic environments. In Figure 13a–c illustrates measurements at UAV heights of 40, 50, and 60 m and their impacts on user throughput distribution for the real measured signal distribution collected and error distribution values. The accuracy of LSTM starts at 0.46 and inclines at around 0.55 MSE with constant variation. The accuracy of MLP starts at around 0.54 and inclines with the LSTM approach at around 0.60 with great variation until the end of the evaluation. However, in Figure 13b, the LSTM accuracy evaluation starts at 0.42 and inclines at around 0.55 before decreasing until the end of the evaluation process, and the MLP accuracy starts at around 0.58 MSE, and its accuracy has the highest variation until the end of the iterations. In Figure 13c, the MSE training error for both LSTM and MLP starts at 0.27 and inclines at around 0.55 and 0.60 with constant variation, but MLP has greater variation than the other iterations until the end of the evaluation. Therefore, the results indicate that the training accuracy of LSTM is better than that of MLP, with an increased number of iterations to manage their great variation and generate a better result. Further, the results indicate that their integration is crucial.

Table 5, shows a comparison of system performances for user throughput maximization using MLP, LSTM, and a hybrid of the MLP-LSTM algorithms within the three classes and UAV heights. When the MLP algorithm was used in class I, the average throughput, MSE, MAPE, and RMSEs at heights of 40, 50, and 60 m were 94.00%, 95.03%, and 93%; 0.004, 0.003, and 0.005; 0.028, 0.027, and 0.031; and 0.5, 0.47, and 0.49, respectively. In the case of LSTM, the average throughput, MSE, MAPE, and RMSEs at heights of 40, 50, and 60 m were 94.00%, 96.33%, and 95.30%; 0.023, 0.033, and 0.031; 0.022, 0.033, and 0.31; and 0.4, 0.36, and 0.37, respectively. For MLP-LSTM, the average throughput, MSE, MAPE, and RMSEs at heights of 40, 50, and 60 m were 96.00%, 98.33%, and 97.30%; 0.01, 0.01, and 0.02; 0.012, 0.013, and 0.011; and 0.29, 0.27, and 0.28, respectively. In class I, the minimum RMSE values were obtained for both MLP and LSTM algorithms, while the hybrid of MLP-LSTM performed better. When the MLP algorithm was used in class II, the average throughput, MSE, MAPE, and RMSEs at heights of 40, 50, and 60 m were 92.00%, 95.51%, and 92%; 0.003, 0.002, and 0.021; 0.020, 0.021, and 0.210; and 0.47, 0.43, and 0.51, respectively. In the case of LSTM, the average throughput, MSE, MAPE, and RMSEs at heights of 40, 50, and 60 m were 95.01%, 97.21%, and 94.08%; 0.022, 0.021, and 0.012; 0.030, 0.029, and 0.028; and 0.32, 0.31, and 0.31, respectively. For the case of MLP-LSTM, the average throughput, MSE, MAPE, and RMSEs at heights of 40, 50, and 60 m were 96.01%, 98.01%, and 95.00%; 0.12, 0.11, and 0.14; 0.011, 0.012, and 0.010; and 0.28, 0.16, and 0.30, respectively. In class-II, the minimum RMSE was obtained for both the MLP and LSTM algorithms, but the MLP-LSTM performance was better for the 50 m height. Using the MLP algorithm in class-III, the average throughput, MSE, MAPE and RMSEs at heights of 40, 50, and 60 m were 92.33%, 95.01%, and 92.15%; 0.034, 0.022, and 0.023; 0.050, 0.041, and 0.030; and 0.45, 0.48, and 0.53, respectively. In the case of LSTM, the average throughput, MSE, MAPE, and RMSEs at heights of 40, 50, and 60 m were 95.53%, 96.75%, and 94.87%; 0.04, 0.03, and 0.02; 0.080, 0.095, and 0.091; and 0.35, 0.38, and 0.33, respectively. For MLP-LSTM, the average throughput, MSE, MAPE, and RMSEs at heights of 40, 50, and 60 m were 96.01%, 97.05%, and 95.97%; 0.03, 0.03, and 0.02; 0.017, 0.018, and 0.019; and 0.28, 0.28, and 0.31, respectively. In class-III, the minimum RMSE values were obtained for both the MLP and LSTM algorithms, but the MLP-LSTM performance was better for the 40 and 50 m heights.

Regarding the throughput maximization of each class distribution, the increment in UAV height from 40 to 50 m led to better user coverage and user throughput distribution in each class. In class I, for example, at UAV heights of 40, 50, and 60 m, RMSE values of 0.29, 0.27, and 0.28 were achieved for throughput, respectively. This indicates that an average distance of 50 m achieves a better result than the other UAV heights. In the case of class II, UAV heights of 40, 50, and 60 m achieved 0.28%, 0.28% and 0.16% throughput, respectively. This shows that an average UAV height of 60 m is better than the other UAV heights.

**Figure 14:**
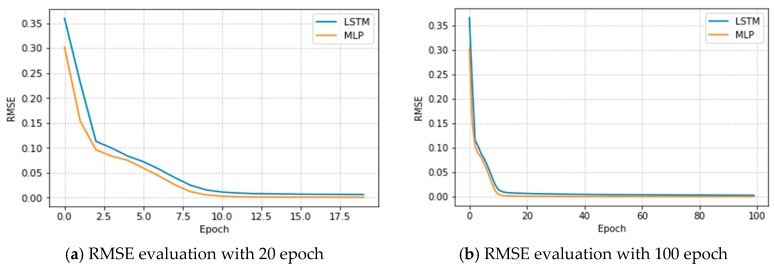
The RMSE over the number of iterations for the LSTM and MLP models.

Regarding class III, UAV heights of 40, 50, and 60 m achieved RMSE values of 0.28, 0.28, and 0.21 throughput, respectively. This shows that the averaged results of both the 40 and 50 m UAV heights were better than those of other UAV heights. Therefore, the MLP-LSTM performs better in both throughput maximization and error distribution for each class and UAV heights. This implies the hybrid of the MLP-LSTM algorithm performs better than using the MLP and LSTM algorithms separately. We also compared the use of the MLP and LSTM algorithms alone with the MLP-LSTM algorithm hybrid and found better results, especially in terms of computing times. The increment in UAV heights does not always have good coverage. For example, at a UAV height of 60 m, the throughput and the error distributions were not as good as those at 50 m. Hence, the placement of the UAV is highly dependent upon the environment that serves as the BS. As a result, the UAV placing at a height of 50 m in sub-urban or urban areas, like our university complex, and elevated buildings is better than others. Figure 14a,b, shows the RMSE estimation values versus the number of iterations for our proposed model. Variation was identified in the UAV signal and throughput measurement error when different numbers of epochs were used. The figure shows the measured results of the MLP and LSTM models and can be used to accurately measure the signal of each grid point and user to show the number of users contained in the UAV coverage. As a result, the proposed system can maximize user throughput with UAV heights and user distances and enhance the coverage capacity and reliability of UAVs. Furthermore, the proposed MLP-LSTM model can accurately measure the user throughput, even when the users are moved.

Table 6 compares the performance of the MLP-LSTM algorithm with the MLP and LSTM algorithms separately in terms of the values of user throughput maximization based on specified scenarios. The training and testing times were used as parameters for comparison. The results illustrate that in terms of the running time, the MLP-LSTM model has shorter training and testing times than the other two models. The training and testing times of MLP, LSTM, and MLP-LSTM in scenarios 1, 2, and 3 were 1.87, 1.47, and 1.52; 1.33, 1.45, and 1.49; 1.11, 1.31, and 1.40; 0.5, 0.47, and 0.49; 0.41, 0.4, and 0.37; and 0.36, 0.31, and 0.34, respectively. 

The performance evaluation showed better training and testing times for user throughput maximization with the MLP-LSTM algorithm. The MLP-LSTM prediction performance values were 1.11, 1.31, and 1.40 for training and 0.36, 0.31, and 0.34 for testing for each scenario, respectively. Therefore, the proposed MLP-LSTM based methods can provide efficient and scalable maximization of UAV positioning based on user throughput in real environments.

## 6. Conclusions and Future Works

In this paper, the MLP-LSTM hybrid algorithm was presented for solving the problem of UAV positioning to maximize user throughput. We used LDA to preprocess the OFDM data sources and selected the strongest values for the training and testing phases. The proposed system was evaluated with respect to classification and regression based on UAV positioning. Our proposed method was also compared with SVM, MLP, and LSTM in different scenarios to evaluate its positioning accuracy. The proposed system was found to have a 98% positioning accuracy with respect to the classification scheme. For regression-based positioning, it achieved accuracy levels of 94.73%, 98.33%, and 99.53% for the selected scenarios. Additionally, our method was found to have a lower error mean, which implied that the error distributions in each set of testing points were low and slow. The computational time complexities in each scenario were also improved under the proposed method compared with other algorithms. Thus, the integration of the MLP-LSTM approach gives state-of-the-art performance on UAV positioning, allowing user throughput maximization on outdoor environments. As a future work, DL will be applied for indoor–outdoor UAV positioning in various environments, such as urban, suburban, and rural areas. 

## Figures and Tables

**Table 1:** Summary of some related works for user throughput maximization.

No.	Author	Title	Objective and Algorithm Used	Result
1	[3]	Positioning of Unmanned Aerial Vehicles (UAVs) for Throughput Maximization in Software-Defined Disaster Area UAV Communication Networks.	To maximize the total throughput by optimal positioning of UAVs using the heuristic method and approximation algorithm.	The average user throughput improvement with the optimal positioning of UAVs was 26%.
2	[4]	UAV Positioning for Throughput Maximization.	Used heuristic and approximation algorithms.	The approximation algorithm gave better results than the heuristic algorithm.
3	[16]	Placement Optimization for UAV-Enabled Wireless Networks with Multi-Hop Backhauls.	To improve the common throughput among all ground users using successive convex programming (SCP).	Showed the effectiveness of common throughput.
4	[31]	Throughput Maximization for Mobile Relaying Systems.	To create optimal power allocations across different time slots using SCP.	Showed the effectiveness of power allocation.
5	[39]	Throughput Maximization in Wireless Powered Communication Networks.	To improve the user throughput and power usage consumption using the hybrid access point (HAP).	Showed the effectiveness of common throughput.
6	[41]	Throughput Maximization for UAV-Enabled Wireless Powered Communication Networks.	To solve the common throughput maximization problem using SCP.	Achieved 80% accuracy.
7	[47]	ML for Predictive On-Demand Deployment of UAVs for Wireless Communications.	To reduce power transmission and improve the throughput using the ML framework, Gaussian mixture model (GMM), and the weighted expectation maximization (WEM) algorithm.	Reduced the power usage and improved the power efficiency by over 20%.
8	Our work	UAV Positioning for Throughput Maximization Using Deep Learning Approaches.	To maximize user throughput with UAV positioning and apply DL approaches with LSTM-MLP, K-means, and IWI distance measurements.	Achieved classification and prediction performance accuracy levels of 94.73, 98.33%, and 99.53% for scenarios 1, 2, and 3, respectively.

**Table 2:** Description of the training and testing datasets.

Data Source	Number of Reference Points (RPs)
Training data	722
Testing data	85
Total	807

**Table 3:** Simulation parameters.

Description	Parameters	Values
Environment (Area)	A	500 m × 300 m
UAV	M	3
Height of UAVs	H	(40, 50, 60 m)
Carrier frequency	f_c_	900 MHz
Output power of transmitter (Tx)	p_t_	29.5 dBm
Signal type	S	OFDM
Number of users	U	35

**Table 4:** Classification accuracy for user throughput evaluation.

	Performance Evaluation		
Algorithm	Scenario 1	Scenario 2	Scenario 3
SVM	80.2%	87.00%	89.1%
MLP	87.23%	92.03%	96.12%
LSTM	94.11%	95.14%	96.21%
MLP-LSTM	96.73%	98.33%	99.53%

**Table 5:** Comparison of user distribution and throughput performance with regression-based algorithms.

		Algorithms											
Class	Height (m)	MLP				LSTM				MLP-LSTM			
		Avg. T. (%)	MSE	MAPE	RMSE	Avg. T. (%)	MSE	MAPE	RMSE	Avg. T. (%)	MSE	MAPE	RMSE
I	40	94.00	0.004	0.028	0.5	94.00	0.023	0.022	0.4	96.00	0.01	0.012	0.29
	50	95.03	0.003	0.027	0.47	96.33	0.002	0.033	0.36	98.33	0.01	0.013	0.27
	60	93.00	0.005	0.031	0.49	95.30	0.031	0.031	0.37	97.30	0.02	0.011	0.28
II	40	92.01	0.003	0.020	0.47	95.01	0.022	0.030	0.32	96.01	0.12	0.011	0.28
	50	95.51	0.002	0.021	0.43	97.21	0.021	0.029	0.31	98.01	0.11	0.012	0.16
	60	92.00	0.021	0.210	0.51	94.08	0.012	0.028	0.31	95.00	0.14	0.010	0.30
III	40	92.33	0.034	0.050	0.45	95.53	0.04	0.080	0.35	96.01	0.03	0.017	0.28
	50	95.01	0.022	0.041	0.48	96.75	0.03	0.095	0.33	97.05	0.03	0.018	0.28
	60	92.15	0.023	0.030	0.53	94.87	0.02	0.091	0.38	95.97	0.02	0.019	0.31

**Table 6:** Comparison of algorithms for performance analysis.

Scenario’s	Algorithms					
	MLP		LSTM		MLP-LSTM	
	Training Time(s)	Testing Time(s)	Training Time(s)	Testing Time(s)	Training Time(s)	Testing Time(s)
1	1.87	0.5	1.33	0.41	1.11	0.36
2	1.47	0.47	1.45	0.4	1.31	0.31
3	1.52	0.49	1.49	0.37	1.40	0.34

## Acronyms

A2A	Air-to-Air
A2G	Air-to-Ground
ANN	Artificial Neural Network
APs	Access Points
BSs	Base Stations
CNN	Convolutional Neural Networks
CSV	Comma-Separated Values
CPU	Central Processing Unit
DL	Deep Learning
5G	Fifth-Generation
GB	Gigabytes
GPS	Global Positioning System
GHz	Gigahertz
G2G	Ground-to-Ground
GRC	GNU Radio Companion
GW	Gateway
HAP	Hybrid Access Point
IoT	Internet-of-Things
IWIP	Iterative Weighted Interpolation Positioning
KNN	K-nearest Neural Network
LDA	Linear Discriminant Analysis
LoS	Line-of-Sight
LSTM	Long Short Term Memory
LTE	Long Term Evolution
MAPE	Mean Absolute Percentage Error
ML	Machine Learning
MSE	Mean Square Error
MHz	Mega Hertz
MLP	Multi-Layer Perceptron
NTUT	National Taiwan University of Technology
NLoS	Non-Line-of-Sight
OFDM	Orthogonal Frequency-Division Multiplexing
PC	Personal Computer
QoS	Quality-of-Service
RAM	Random Access Memory
ReLU	Rectified Linear Unit
RPs	Reference Points
RNN	Recurrent Neural Network
RMSE	Root Mean Square Error
RSS	Received Signal Strength
Rx	Receiver
SDN	Software-Defined Network
SNR	Signal-Noise-Ratio
Tx	Transmitter
UAV	Unmanned Aerial Vehicles
UE	User Equipment
U2U	User-to-User
USRP	Universal Software Radio Peripheral
Wi-Fi	Wireless Fidelity
WPCN	Wireless Powered Communication Networks
3D	Three-Dimensional

## References

[1] Cai G., Dias J., Seneviratne L. (2014). A Survey of Small-Scale Unmanned Aerial Vehicles: Recent Advances and Future Development Trends. Unmanned Syst..

[2] Yahyanejad S., Wischounig-Strucl D., Quaritsch M., Rinner B. Incremental Mosaicking of Images from Autonomous, Small-Scale UAVs. Proceedings of the 7th IEEE International Conference on Advanced Video and Signal Based Surveillance (AVSS 2010).

[3] Rahman S.U., Kim G.-H., Cho Y.-Z., Khan A. (2018). Positioning of UAVs for throughput maximization in software-defined disaster area UAV communication networks. J. Commun. Netw..

[4] Rahman S.U., Cho Y.-Z. (2018). UAV positioning for throughput maximization. EURASIP J. Wirel. Commun. Netw..

[5] Mozaffari M., Saad W., Bennis M., Debbah M. (2016). Efficient Deployment of Multiple Unmanned Aerial Vehicles for Optimal Wireless Coverage. IEEE Commun. Lett..

[6] Mozaffari M., Saad W., Bennis M., Debbah M. Drone Small Cells in the Clouds: Design, Deployment and Performance Analysis. Proceedings of the IEEE Global Communication Conference (GLOBECOM).

[7] De Freitas E.P., Heimfarth T., Vinel A., Wagner F.R., Pereira C.E., Larsson T. (2013). Cooperation among wirelessly connected static and mobile sensor nodes for surveillance applications. Sensors.

[8] Bekmezci I., Sahingoz O.K., Temel Ş. (2013). Flying Ad-Hoc Networks (FANETs): A survey. Ad Hoc Netw..

[9] Mozaffari M., Saad W., Bennis M., Debbah M. (2016). Unmanned Aerial Vehicle with under Laid Device-to-Device Communications: Performance and Tradeoffs. IEEE Trans. Wirel. Commun..

[10] Orfanus D., De Freitas E., Eliassen F. (2016). Self-Organization as a Supporting Paradigm for Military UAV Relay Networks. IEEE Commun. Lett..

[11] Košmerl J., Vilhar A. Base Stations Placement Optimization in Wireless Networks for Emergency Communications. Proceedings of the IEEE International Conference on Communication (ICC).

[12] Balanis C.A. (2016). Antenna Theory: Analysis and Design.

[13] Al-Hourani A., Kandeepan S., Jamalipour A. Modeling Air-to-Ground Path Loss for Low Altitude Platforms in Urban Environments. Proceedings of the IEEE Global Communication Conference (GLOBECOM).

[14] Zeng Y., Zhang R., Lim T.J. (2016). Wireless communications with unmanned aerial vehicles: Opportunities and challenges. IEEE Commun. Mag..

[15] Wu Q., Zhang R. (2018). Common Throughput Maximization in UAV-Enabled OFDMA Systems with Delay Consideration. arXiv.

[16] Li P., Xu J. (2018). Placement optimization for UAV-enabled wireless networks with multi-hop backhauls. arXiv.

[17] Zhang C., Zhang W. (2017). Spectrum sharing for drone networks. IEEE J. Sel. Areas Commun..

[18] Li P., Xu J. (2017). Placement optimization of UAV-mounted mobile base stations. IEEE Commun. Lett..

[19] He H., Zhang S., Zeng Y., Zhang R. (2018). Joint altitude and beam width optimization for UAV-enabled multiuser communications. IEEE Commun. Lett..

[20] Yaliniz R.I.B., El-Keyi A., Yanikomeroglu H. Efficient 3-D Placement of an Aerial Base Station in Next Generation Cellular Networks. Proceedings of the IEEE International Conference on Communications.

[21] Chen J., Yatnalli U., Gesbert D. Learning Radio Maps for UAV-Aided Wireless Networks: A Segmented Regression Approach. Proceedings of the IEEE International Conference on Communications.

[22] Zeng Y., Xu X., Zhang R. (2018). Trajectory design for completion time minimization in UAV-enabled multicasting. IEEE Trans. Wirel. Commun..

[23] Wu Q., Zeng Y., Zhang R. (2018). Joint trajectory and communication design for multi-UAV enabled wireless networks. IEEE Trans. Wirel. Commun..

[24] Han Z., Swindlehurst A.L., Liu K.J.R. (2009). Optimization of MANET connectivity via smart deployment/movement of unmanned air vehicles. IEEE Trans. Veh. Technol..

[25] Kim S., Oh H., Suk J., Tsourdos A. (2014). Coordinated trajectory planning for efficient communication relay using multiple UAVs. Control. Eng. Pract..

[26] Johansen T.A., Zolich A., Hansen T., Sørensen A.J. Unmanned Aerial Vehicle as Communication Relay for Autonomous Underwater Vehicles—Field Tests. Proceedings of the IEEE Global Communication Conference (GLOBECOM).

[27] Yong Z., Zhang R., Lim T.J. (2016). Throughput maximization for UAV-enabled mobile relaying systems. arXiv.

[28] Sirait K., Tulus T., Nababan E. (2017). K-Means algorithm performance analysis with determining the value of starting centroid with random and KD-tree method. J. Phys. Conf. Ser..

[29] Ren H., Song Y., Liu J., Hu Y., Lei J. (2017). A Deep Learning Approach to the Prediction of Short-Term Traffic Accident Risk. arXiv.

[30] Abdelhadi A., Pujolle G. (2017). A Long Short-Term Memory Recurrent Neural Network Framework for Network Traffic Matrix Prediction. arXiv.

[31] Umeh O.A., Akpado K.A., Okechukwu G.N., Ejiofor H.C. (2015). Throughput and delay analysis in a real time network. Int. J. Eng. Appl. Sci. (IJEAS).

[32] Adege A.B., Lin H.-P., Tarekegn G.B., Munaye Y.Y., Yen L. (2018). An indoor and outdoor positioning using a hybrid of support vector machine and deep neural network algorithms. J. Sens..

[33] Hochreiter S., Schmidhuber J. (1997). Long short-term memory. Neural Comput..

[34] Sundermeyer M., Ney H., Schlüter R. (2015). From Feedforward to Recurrent LSTM Neural Networks for Language Modeling. IEEE Trans. Audio Speech Lang. Process..

[35] Ju H., Zhang R. (2014). Throughput Maximization in Wireless Powered Communication Networks. IEEE Trans. Wirel. Commun..

[36] Liu L., Zhang R., Chua K.-C. (2014). Multi-Antenna Wireless Powered Communication with Energy Beamforming. IEEE Trans. Commun..

[37] Park J., Lee H., Eom S., Lee I. (2018). Minimum Throughput Maximization in UAV-Aided Wireless Powered Communication Networks. arXiv.

[38] Barritt B., Kichkaylo T., Mandke K., Zalcman A., Lin V. Operating a UAV Mesh & Internet Backhaul Network Using Temporospatial SDN. Proceedings of the IEEE Aerospace Conference.

[39] Secinti G., Darian P.B., Canberk B., Chowdhury K.R. (2018). SDNs in the sky: Robust end-to-end connectivity for aerial vehicular networks. IEEE Commun. Mag..

[40] Zhao N., Cheng F., Yu F.R., Tang J., Chen Y., Gui G., Sari H. (2018). Caching UAV Assisted Secure Transmission in Hyper Dense Networks Based on Interference Alignment. IEEE Trans. Commun..

[41] Bor-Yaliniz I., Yanikomeroglu H. (2016). The new frontier in RAN heterogeneity: Multi-tier drone-cells. IEEE Commun. Mag..

[42] Cheng F., Zhang S., Li Z., Chen Y., Zhao N., Yu F.R., Leung V.C.M. (2018). UAV trajectory optimization for data offloading at the edge of multiple cells. IEEE Trans. Veh. Technol..

[43] Rosário D., Filho J.A., Rosário D., Santosy A., Gerla M. A Relay Placement Mechanism Based on UAV Mobility for Satisfactory Video Transmissions. Proceedings of the 2017 16th Annual Mediterranean Ad Hoc Networking Workshop (Med-Hoc-Net).

[44] Zhan P., Yu K., Swindlehurst A.L. (2011). Wireless relay communications with unmanned aerial vehicles: Performance and optimization. IEEE Trans. Aerospace Electron. Syst..

[45] Dixon C., Frew E.W. (2012). Optimizing cascaded chains of unmanned aircraft acting as communication relays. IEEE J. Sel. Areas Commun..

[46] Kalantari E., Shakir M.Z., Yanikomeroglu H., Yongacoglu A. Backhaul-Aware Robust 3D Drone Placement in 5G+ Wireless Networks. Proceedings of the 2017 IEEE International Conference on Communications Workshops (ICC Workshops).

[47] Liu S., Wei Z., Guo Z., Yuan X., Feng Z. Performance Analysis of UAVs Assisted Data Collection in Wireless Sensor Network. Proceedings of the 2018 IEEE 87th Vehicular Technology Conference (VTC Spring).

[48] Lee K., Kim J.K., Kim J., Hur K., Kim H. CNN and GRU Combination Scheme for Bearing Anomaly Detection in Rotating Machinery Health Monitoring. Proceedings of the 1st IEEE International Conference on Knowledge Innovation and Invention.

[49] Graves A. (2012). Supervised Sequence Labelling with Recurrent Neural Networks.

[50] Goodfello I., Bengio Y., Courville A. (2016). Deep Learning.

[51] Zhang Q., Mozaffari M., Saad W., Bennis M., Debbah M. Machine learning for predictive on-demand deployment of UAVs for wireless communications. Proceedings of the 2018 IEEE Global Communications Conference (GLOBECOM).

[52] Xuan S., Kanasugi H., Shibasaki R. Deep Transport: Prediction and Simulation of Human Mobility and Transportation Mode at a Citywide Level. Proceedings of the 25th International Joint Conference on Artificial Intelligence (IJCAI).

